# Distinct Mechanisms in Number Comparison of Random and Regular Dots: An ERP Study

**DOI:** 10.3389/fnbeh.2021.791289

**Published:** 2022-01-12

**Authors:** Wei Liu, Yajun Zhao, Chunhui Wang, Lu Wang, Ying Fu, Zhijun Zhang

**Affiliations:** ^1^College of Education, Dali University, Dali, China; ^2^Department of Psychology and Behavioral Sciences, Zhejiang University, Hangzhou, China; ^3^School of Education, Yunnan Minzu University, Kunming, China; ^4^School of Education and Psychology, Southwest Minzu University, Chengdu, China

**Keywords:** numerosity perception, density perception, P2p components, element distribution, distance effect

## Abstract

Numerosity comparison for regular patterns shows different features compared with that for random ones in previous studies, suggesting an underlying mechanism distinct from numerosity. In this study, we went further to compare the event-related potentials (ERP) components in numerosity processing of random and regular patterns, which are identical in all aspects of texture features except for the distribution. ERP components were recorded and analyzed while participants compared which of the two successively presented sets was more numerous. P2p amplitude was revealed to be significantly weaker for regular patterns compared with that for random patterns over right occipital-parietal cites, whereas no difference was found for P1 or N1 components. The difference in P2p amplitude, which is consistent with the behavior dissociation revealed in our previous studies, suggests that regular distribution can trigger distinct processing in numeral comparison tasks. Processing of continuous magnitudes or configuration cannot explain the decrease in P2p amplitude for regular distributed patterns. Therefore, this study further supports that P2p is mediated by numerosity processing.

## Introduction

The ability to process numerosity enables us to rapidly enumerate, generate, and compare numeral information (Dehaene, 2002; Cantlon and Brannon, 2005; Cicchini et al., 2016, 2019). The perception of numerosity emerges with the analysis of texture features; therefore, it is difficult to dissociate numerosity processing from continuous magnitude processing. Some researchers suggested that numerosity information can be appraised spontaneously. Observers are far more sensitive to numerosity information rather than other texture information such as area or density when they were simply told to detect any change of two sets of stimuli, even if the dimension of numerosity was not emphasized (Cicchini et al., 2016, 2019). However, others hold the idea that numerosity information is a coproduct, which is inferred from the processing of continuous magnitudes such as density and area (Raphael and Morgan, 2015; Durgin, 2017; Yousif and Keil, 2020).

Distinct mechanisms can underlie the number comparison tasks within different number ranges. As for small numbers 1–4, a mechanism labeled subitizing enables us to process numbers with extremely high efficiency (Kaufman and Lord, 1949). Anobile et al. (2014) found that when dot density goes beyond the moderate range, Weber fractions for the number comparison task change their mode. Within moderate number range, constant Weber fractions indicate the activity of numerosity or approximate number system (ANS) mechanism, whereas when stimuli become denser, Weber fractions decrease linearly with the reference number, suggesting a mechanism based on density (Anobile et al., 2014). According to the study of Fornaciai and Park (2017), the sensitivity of visual-evoked potentials (VEPs) to large numerosity (400 dots) was found in the later latency point (180 ms), which is stronger compared with the sensitivity to size and spacing, consistent with the results concerning ANS range (Park et al., 2016). Importantly, the sensitivity for large numerosity is weaker than that for ANS or moderate number range, suggesting that different sources of information might concur in the formation of a numerosity representation at the early stages of visual processing (Fornaciai and Park, 2017).

As density increases, it becomes more difficult to separate individual dots (i.e., crowding-like effect), and the processing of individuation can be inhibited (Anobile et al., 2015). The processing of individuation can be demonstrated by investigating connectedness effects, which refers to the phenomenon that two dots are taken as “one” when they are connected by a line and that connection induces underestimation (He et al., 2009; Anobile et al., 2017). Inhibition of individuation is accompanied by inhibition of generic numerosity processing, which refers to the processing underlying numeral tasks with a moderate number of randomly distributed stimuli (Dehaene, 1996; Halberda et al., 2008; Anobile et al., 2014). Processing features that are typically revealed in these tasks can be absent when individuation is interrupted (Liu et al., 2017, 2018). Besides the crowding-like effect (Anobile et al., 2015), dot distribution can also disturb generic numeral processing. For high-regularly distributed patterns, in which dots were arranged in metric, the perception of numerosity is proposed to be different from that for random patterns. First, the effect of connectedness is much weaker for regular patterns, suggesting that individuation is inhibited, probably because the structure of “whole” is emphasized by the highly regular pattern. Second, changes in stimulus orientation and size, although having no effect on adaptation for random patterns, significantly affect the numeral adaptation in regular patterns. Third, the adaptation of regular patterns is monocular transferring (adaptation aftereffects stay in the exposed eye), whereas the adaptation of random adaptor is binocular transferring (adaptation aftereffect can transfer to the unexposed eye (Liu et al., 2017, 2018). Generic numerosity processing seems to be superseded by a distinct mechanism in the coding of regular patterns.

In this study, we went further to compare the event-related potentials (ERP) components in numerosity processing of random and regular patterns. ERP components such as P1, N1, and P2p were recorded and analyzed while participants compared which of the two successively presented sets were more numerous. P1 is activated from 70 to 90 ms and peaking at 80–130 ms after the onset of stimuli and is related to the cluster and location of the stimulus (Anllo-Vento and Hillyard, 1996; Nikolaev et al., 2008). P1 component is supposed to be involved in the early processing of input information when observers notice and process the target stimuli (Mangun, 1995). N1, a negative component peaking at around 150 ms after the onset of stimuli, is proposed to be related to numeral format, stimulus size, and the distributing and maintaining of spatial attention (Hillyard and Anllo-Vento, 1998; Hyde and Spelke, 2012). It is proposed that the change in N1 can further affect P2 and P2p components (Libertus et al., 2007).

P2p is a positive component that peaks at 200–300 ms after the onset of the target. It is generally proposed to highly correlate with numerosity processing. For example, P2p in the parietal-occipital lobe and the intraparietal sulcus (IPS) is related to numerosity representation, estimation, and comparison (Dehaene, 1996; Pinel et al., 2001). Number distance effect, the phenomenon that numerical processing efficiency depends on the ratio of two numbers being compared (Moyer and Landauer, 1967; Libertus et al., 2007; Holloway and Ansari, 2009), is also demonstrated in ERP study (Verguts and Van Opstal, 2005; Libertus et al., 2007; Hyde and Spelke, 2009, 2012). In the occipital-temporal lobe, greater P2p amplitude is revealed in conditions with a shorter distance between reference and test stimulus in the comparison task. Distance effect is found for P2p amplitude in IPS both for adults and 4-year old children, and this effect is proved to be independent of the change in the shape of the stimulus (Piazza et al., 2004; Cantlon et al., 2006).

Some researchers argued that P2p is mediated by the processing of continuous magnitudes such as area or density, rather than the discrete number of stimuli, as these magnitudes always vary with numerosity in previous studies (Gebuis and Reynvoet, 2013). One direct way to demonstrate the sensitivity of the ERP component to numerosity processing is to control every non-numerical visual feature while manipulating numerosity, which is nearly impossible (Leibovich et al., 2017; van Rinsveld et al., 2020). From a new perspective, recent studies tested the effect of numerical and non-numerical cues on the VEPs by a linear model, revealing strong neural sensitivity to numerosity around 160–180 ms over right occipito-parietal cites (Fornaciai and Park, 2017). In addition, as was suggested by another study using a frequency-tagging electroencephalogram (EEG) approach, electrophysiological responses were found to synchronize with the frequency of a periodically occurring deviant numerosity of stimulus (van Rinsveld et al., 2020).

In this study, ERP components for number comparison in random and regular patterns were recorded and compared. To this end, we found that P2p amplitude is significantly weaker for regular patterns compared with that for random patterns over the right occipital-parietal region, which is highly correlated with non-symbolic approximate numeral processing (Piazza et al., 2007; Holloway et al., 2010; Fornaciai and Park, 2017). The difference in P2p amplitude is consistent with the behavior dissociation revealed in previous studies (Liu et al., 2017, 2018), which suggests that regular distribution can trigger a processing mechanism other than numerosity in comparison tasks. With regular stimuli, the sensitivity of observers to numerosity information is significantly lower than that of random stimuli. The current results suggest that the decrease in P2p sensitivity is synchronous with the inhibition of numerosity mechanism in numerical comparison of regular patterns. It is worth noting that regular and random patterns are identical in all aspects of continuous magnitudes, and processing of non-numerical magnitudes cannot explain the decrease in P2p amplitude for regularly distributed patterns. Moreover, the perception of configuration or other attributes such as spatial frequency is unlikely to contribute to the P2p difference in the right occipital-parietal lobe, either. Therefore, this study provides further evidence supporting that P2p is mediated by numerosity processing.

## Materials and Methods

### Statement

For all experiments, all administered measures and tested experimental conditions were reported. Recorded data from 18 participants were all included in the calculation. Missing data (responses beyond 1,000 ms in the responding window) were subtracted from the total response when the selection probability for PSE was calculated. For each participant, missing data were less than 3%.

### Ethics Statement

For all experiments, the data were analyzed anonymously. Participants provided their informed consent in both verbal and written forms, and they were compensated for their participation. The Ethics Committee of the Zhejiang University approved this study.

### Participants

With an α error probability of 0.05 and a power of 0.8, power analysis using G*power 3.1 showed that at least seven participants were needed to yield an effect size of 0.4 in repeated measures ANOVA. Twenty adults (age range = 20–30 years; 10 males) participated in Experiment 1, and two of them were excluded because their ERP data were abnormal. Eventually, there were eighteen participants (9 males; average age = 24.13 years). The adults in all experiments of this study were right-handed, with either normal or corrected-to-normal vision.

### Apparatus

The stimuli were presented on a 19′′ flat-screen monitor with a resolution of 1,366 × 768 pixels and a refresh rate of 100 Hz in a dark room. The monitor was located at approximately 50 cm from the seated participant. All the stimuli were displayed using E-prime 2.0 (PST, Sharpsburgh, PA, United States) software. While viewing stimuli, the ongoing EEG was recorded from 32 channels using a Geodesic Sensor Net digitally filtered at 0.5–100 Hz with an A/D sample rate of 500 Hz. Neuronscan ERP system with a SynAmps amplifier, an Ag/Ag Cl electrode cap, and a 19′′ flat-screen monitor was adopted to record neuron data.

### Data Collection and Analysis

SCAN 4.4 (Neuroscan, Victoria, Australia) was adopted to record and analyze the data. ERP between trials was taken as a baseline for correction. Test trials were segmented into experimental conditions based on 200 ms of recording before to 800 ms of recording after each stimulus presentation. Later, recordings were low-pass filtered at 30 Hz/24 dB. Segments containing artifacts (i.e., eye blink, eye movement, head movement, or excessive noise) and/or beyond the range (−75 μV, 75 μV) were detected and removed using the independent component analysis (ICA). Data with a reject ratio beyond 25% were abandoned. To this end, the data of two participants were excluded. Other artifact-free trials were averaged for each of the experimental conditions and each participant, re-referenced to the average reference, and corrected to 200 ms before stimulus onset. Grand means for each of the experimental conditions and grand averages of small and large number processing were computed for analysis and visualization purposes.

### Scalp Event-Related Potentials Analysis

Three components of interest were targeted, namely, P1, N1, and P2p. Referring to previous literature (Ansari et al., 2006; Piazza et al., 2007), the parietal and the occipital lobe were included in this study. Data from C3, C4, Cz, Cp1, Cp2, Cp5, Cp6, Poz, P3, Pz, P4, O1, O2, and Oz electrodes were recorded. Based on the related studies (Hyde and Spelke, 2012), grand means of eleven electrodes in the parietal lobe and grand averages of three electrodes in the occipital lobe were computed.

### Stimuli

**FIGURE 1:**
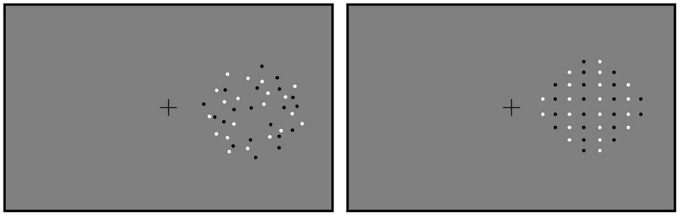
The stimuli used in Experiment 1. Reference in the random and the regular conditions are shown. Reference and test patterns were displayed successively in the hemifield. Therefore, the paths on the other side were blank. The positions of references and tests were counterbalanced across participants.

**FIGURE 2:**
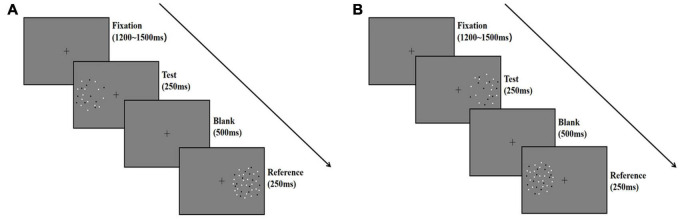
Schematic illustration describing the procedure of Experiment 1. Each trial began with a fixation for 1,200–1,500 ms at random, and then the test stage began with a test stimulus displayed on one side of the screen for 250 ms, followed by a reference stimulus displayed on the other side for 250 ms. The two stimuli were separated by a blank screen lasting 500 ms. Participants fixed their gaze on the fixation point during the trial and reported which pattern (left or right) appeared to be more numerous; they guessed when they were unsure. **(A)** The test stimuli was presented on the left side. **(B)** The test stimuli was presented on the right side.

Two circular patterns of dots were produced using Walk Script 1.0 (ZJU Walkinfo Co., Ltd., Hangzhou, China). One pattern served as a reference and the other as a test. Both patterns were displayed within two fixed circles centered at 7.92° in the middle of the computer screen (Figure 1) in succession (Figure 2). Each gray-scale pattern (RGB: 128, 128, 128) had a diameter of 11.9° and was presented against a gray (RGB: 128, 128, 128) background. Reference and test patterns were classified into two conditions. In Condition 1 (the random condition), dots were randomly distributed in reference and test patterns. In Condition 2 (the regular condition), dots were regularly distributed. Black and white dots were arranged into vertical queues. Black and white queues were composed of dots that were presented in turn from left to right. For each condition, reference patterns were first created, each with 40 square dots (20 were white and 20 were black) with a diameter of 0.4°. Dots in the test patterns were essentially the same (other than the numbers of dots) as those in the reference patterns in each condition. Namely, there were two blocks of experiments for each participant, one is for random pattern and the other is for the regular pattern.

The number of dots in the test patterns varied based on the reference number of 40 in each group. The quantities were chosen using a logarithmic scale. Moreover, we chose a number with which a symmetric pattern could be constructed in regular groups; thus, the tests contained 24, 33, 36, 40, 44, 49, 58, or 68 dots. Tests 24, 36, and 49 were marked, and their EEG signal was recorded and analyzed in each block. Each of these tests was repeated for 100 trials in a random sequence. The ratios between test and reference for these three tests are 0.6 (24/40), 0.9 (36/40), and 0.81 (40/49), respectively, and it is hypothesized that the P2p amplitude depends on the ratio between each two numbers to be compared. Meanwhile, the rest tests were also shown with the chosen ones in the same blocks. Each of the rest tests was only presented in random sequence for 50 trials so that the duration for each experiment is still appropriate. In general, a typical paradigm of constant stimuli method was adopted, in which the participants were asked to compare the reference with a serial of tests.

According to the study of Anobile et al. (2015), the switching point from numerosity to density mechanism is centered at 40 dots (0.8 dots/°^2^, for reference) at 15° eccentricity with a test serial. In this study, stimuli were presented at 7.92° from the center. The reference contained 40 dots (0.36 dots/°^2^), and the 68-dot test was the densest pattern (0.61 dots/°^2^). These parameters ensured that none of the stimulus used was in the density regime; therefore, the comparison task in the random group should activate generic numerosity processing within the ANS range.

### Procedure

The testing stage used a point of subjective equality (PSE) and a just noticeable difference (JND) to quantify numerosity perception. A 2AFC (temporal) task was adopted to produce a psychometric function (dependent variable: probability of test > reference) from which the PSE and JND were extracted as measures of perception. Two sets of dots were successively shown in the two fixed circles in the horizontal direction, and each trial was presented to all the participants with a forced-choice question: “Which circle contained more dots?” Participants pressed buttons to respond: “f” with their left hand denoted that the left circle contained more dots, whereas “j” with their right hand indicated that the right circle was more numerous.

The procedure is described in Figure 2. We introduced the procedure for the random condition as an example. Participants initiated the experiment by pressing the space bar. A fixation point was shown in the center of the screen for 1,200–1,500 ms (randomly varied between trials), followed by the left circle with the test stimuli for 250 ms. Then, the right circle with a reference stimulus was shown for 250 ms. A blank frame with a fixation cross isolated the test and the reference for 500 ms. The next trial began either after the participants responded or after 1,000 ms. The test and reference positions were counterbalanced across participants (Figures 2A,B).

At the beginning of the experiment, a 10-min practice with feedback was conducted to improve the familiarity of participants with the experiment. Then, the participants completed the formal experiment. Tests 24, 36, and 49 were presented for 300 trials (100 trials for each test), and the rest five tests were presented for 250 trials (50 trials for each test). Overall, 550 trials were conducted for each pattern, with sufficient rest between blocks to avoid fatigue.

### Statistical Analysis

Individual PSE, JND, and Weber fraction were calculated to estimate the accuracy and precision of comparison, and 95% CIs were reported. Error rate and reaction time were calculated to estimate the efficiency of comparison. In this study, one-sample *t*-tests were one-tailed, and paired *t*-tests were two-tailed. Cohen’s *d* was reported to provide a complement to null hypothesis statistical significance testing by estimating the magnitude of the difference (0.2–0.5 for small effect size, 0.5–0.8 for moderate effect size, and >0.8 for large effect size). Bayes factors (BF_10_) were reported to estimate whether the null hypothesis H_0_ or the alternative hypothesis H_1_ is more likely to be correct. BF_10_ < 0.3 suggests clear evidence for H_0_, whereas BF_10_ > 3 indicates clear evidence for H_1_.

(Bayes) repeated-measures ANOVA was conducted for both behavioral and ERP data, and η*_*p*_^2^* was reported to estimate the effect of independent variables. Bonferroni correction of *post hoc* test (*P*_*bonf*_) was adopted in the current analyses of multiple comparisons by JASP and SPSS 16. Linear regression was conducted with P2p amplitude as the dependent variable and ratio as the independent variable. Two variants of models were compared, one without intercept and the other with intercept, performing the *F*-test in Origin 9 (OriginLab, Northampton, MA, United States).

## Results

### Behavioral Results

Cumulative normal models were fitted to the psychometric functions of each participant using the psignifit toolbox version 2.5.41 of MATLAB (MathWorks, Natick, MA, United States)^1^ to implement the maximum likelihood method described by Wichmann and Hill (2001) and, thereby, to quantitatively measure the accuracy and precision of the numerosity perception of participants. The 50% points of the fitted functions (parameter of α) were obtained. The values of the test stimuli (abscissa) corresponding to the 50% points were calculated from the fitted curves. These values are the PSEs representing the number of test dots that appeared as equal to the number of reference dots, according to the participants. JND of the comparison can be measured by the width of the fitted function (parameter of β). Weber fraction (*W*) is equal to the division between JND and PSE (Anobile et al., 2014). For the random group, averaged PSE = 42.68 (SD = 4.44), 95% CI = [40.41, 44.95] and averaged *W* = 0.33 (SD = 0.22), 95% CI = [0.22, 0.45]. For the regular group, averaged PSE = 46.49 (SD = 6.84), 95% CI = [42.99, 49.99] and averaged *W* = 0.34 (SD = 0.21), 95% CI = [0.23, 0.45]. Paired *t-*test showed no significant difference for Weber fractions, *t*_(17)_ = 1.035, *p* = 0.315, Cohen’s *d* = 0.244, BF_10_ = 0.388.

**FIGURE 3:**
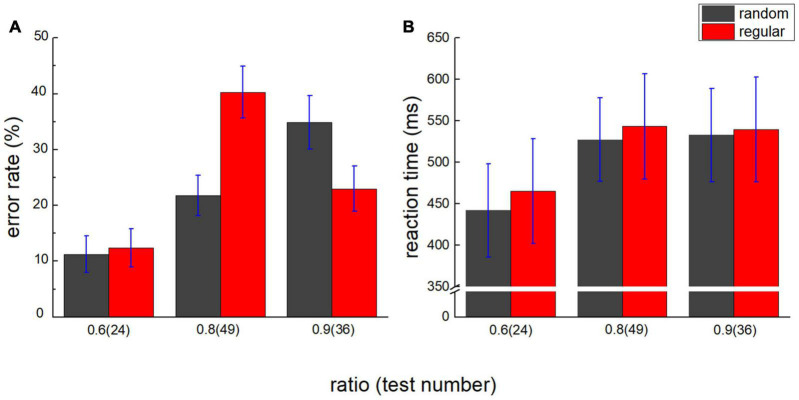
Number distance effect on error rate and reaction time. **(A)** Error rate of test 24 (ratio = 0.6), 49 (ratio = 0.8), and 36 (ratio = 0.9) is plotted against the number ratio. Black bars denote the random group, and red bars stand for the regular group. **(B)** Reaction time is plotted against the number ratio. Number distance effect is suggested in both groups. Error bars denote 1 SE.

The error rate of comparison was plotted against the ratio in Figure 3A, and the reaction time was plotted in Figure 3B. A 2 (stimuli type: random/regular) × 3 (number ratio: 0.6/0.8/0.9) repeated measures ANOVA with the error rate as dependent variable was conducted. The main effect of the stimuli type is not significant, *F*_(1,17)_ = 0.425, *p* = 0.523, η*_*p*_^2^* = 0.024, and BF_10_ = 0.244. The main effect of the ratio is significant, *F*_(2,34)_ = 12.775, *p* < 0.001, η*_*p*_^2^* = 0.429, BF_10_ > 100. *Post hoc* tests show the difference in the error rate between ratio 0.6 and 0.8, *P*_*bonf*_ = 0.001, and between ratio 0.6 and 0.9, *P*_*bonf*_ < 0.001. No significant difference is suggested between ratio 0.8 and 0.9, *P*_*bonf*_ = 1.000. The number distance effect on the error rate is suggested. Significant interaction is revealed, *F*_(2,34)_ = 8.142, *p* = 0.001, η*_*p*_^2^* = 0.324, and BF_10_ > 100. In the random group, *post hoc* tests show the difference between ratio 0.6 and 0.9, *t*_(17)_ = 5.078 and *P*_*bonf*_ < 0.001. No significant difference is suggested between ratio 0.8 and 0.9, *t*_(17)_ = 2.928 and *P*_*bonf*_ = 0.070, or between ratio 0.6 and 0.8, *t*_(17)_ = 2.150, *P*_*bonf*_ = 0.527. In the regular group, *post hoc* tests show the difference between ratio 0.6 and 0.8, *t*_(17)_ = 4.017 and *P*_*bonf*_ < 0.001. No significant difference is suggested between ratio 0.6 and 0.9, *t*_(17)_ = 1.158 and *P*_*bonf*_ = 1.000, or between ratio 0.8 and 0.9, *t*_(17)_ = 2.499 and *P*_*bonf*_ = 0.223.

A 2 (stimuli type: random/regular) × 3 (number ratio: 0.6/0.8/0.9) repeated measures ANOVA with reaction time as dependent variable revealed a similar number distance effect. The main effect of the stimuli type is not significant, *F*_(1,17)_ = 0.415, *p* = 0.528, η*_*p*_^2^* = 0.024, and BF_10_ = 0.284. The main effect of the ratio is significant, *F*_(2,34)_ = 13.891, *p* < 0.001, η*_*p*_^2^* = 0.450, and BF_10_ > 100. *Post hoc* tests show the difference in the error rate between ratio 0.6 and 0.8, *P*_*bonf*_ < 0.001, and between ratio 0.6 and 0.9, *P*_*bonf*_ = 0.002. No significant difference is suggested between ratio 0.8 and 0.9, *P*_*bonf*_ = 1.000. No significant interaction is revealed, either, *F*_(2,34)_ = 0.217, *p* = 0.806, η*_*p*_^2^* = 0.013, and BF_10_ = 0.162.

### Event-Related Potentials Components

**FIGURE 4:**
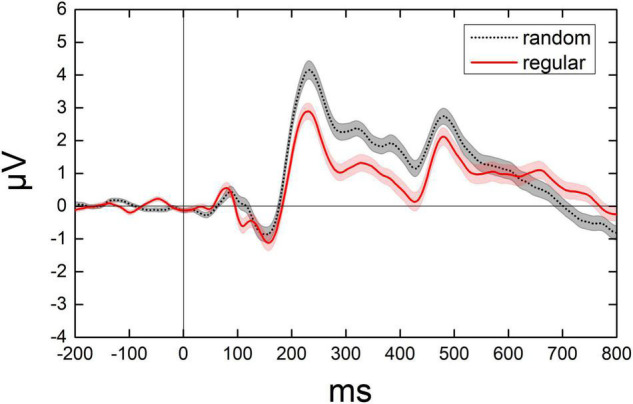
Illumination for the dynamic change of average ERPs from 14 electrodes. The average value of average ERPs of participants from 200 ms before to 800 ms after each test. Black curves denote the ERPs when randomly distributed dots were presented. Red curves denote the ERPs when the regularly distributed dots were presented. Shadowed area denotes 1 SE of the mean.

**FIGURE 5:**
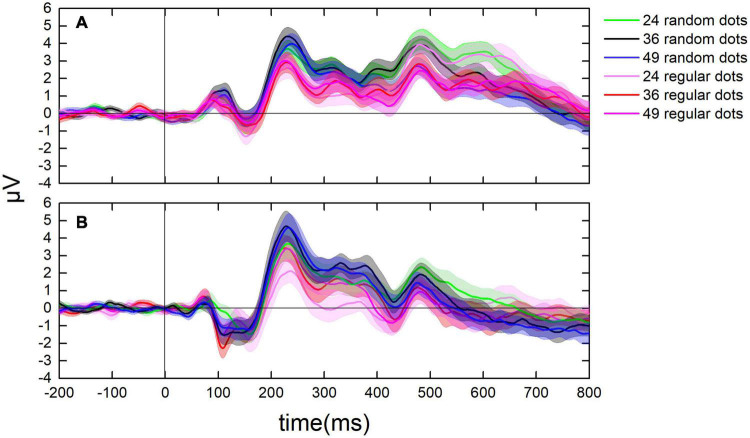
Dynamic change of ERPs in the parietal and the occipital lobes. **(A)** The average value of ERPs of participants in the parietal lobe from 200 ms before to 800 ms after each test. **(B)** The ERPs in the occipital lobe from 200 ms before to 800 ms after each test. Curves in cool colors denote the ERPs when randomly distributed tests were presented, whereas curves in warm colors stand for the ERPs when regularly distributed tests were presented.

After the onset of the test, a positive component (P1) showed up in the parietal lobe with a peak latency at around 90 ms, followed by a negative component (N1) in the parietal-occipital lobe with a peak latency at around 150 ms, and a posterior positive component (P2p) in the parietal-occipital lobe with a peak latency at around 225 ms. Figure 4 shows the dynamic change of the average ERPs of occipital and parietal lobes (from 14 electrodes). Figure 5 demonstrates the ERPs for distinct test ratios and patterns separately in different colors.

### P1 Component

**FIGURE 6:**
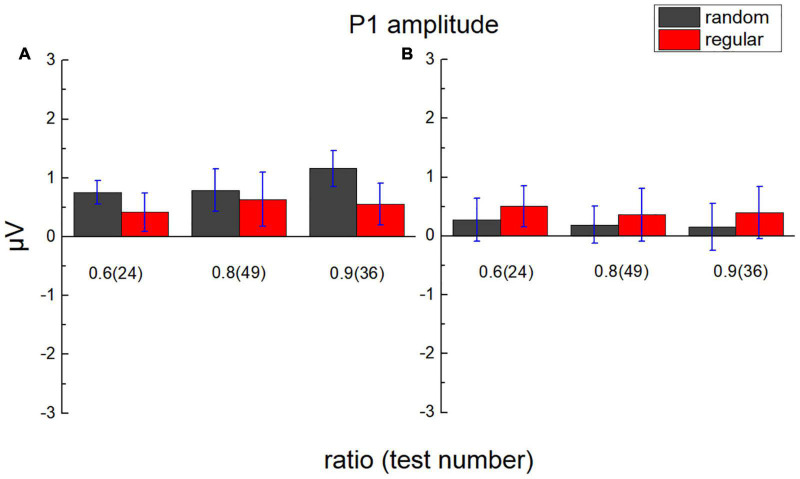
Average P1 amplitude in the parietal lobe and the occipital lobe. **(A)** The average amplitude of the ERP of participants in the parietal lobe from 80 to 120 ms after test presentation (P1) is demonstrated according to different test ratios (0.6, 0.8, and 0.9). **(B)** The average amplitude of ERP in the occipital lobe from 65 to 90 ms after the test. Black bars stand for the randomly distributed patterns, and red ones stand for the regularly distributed patterns. Error bars denote 1 SE of the mean.

The average value of the ERP amplitude of participants in the parietal lobe from 80 to 120 ms and the average ERP amplitude in the occipital lobe from 65 to 90 ms after each test were calculated as average P1 amplitude (Figure 6). The average ERP amplitude is significantly greater than “0,” *t*_(17)_ = 1.948, *p* = 0.034, Cohen’s *d* = 0.459, and BF_10_ = 2.178.

A 2 (lobe: parietal/occipital) × 2 (stimulus type: regular/random) × 3 (test ratio: 0.6/0.8/0.9) repeated measures ANOVA was adopted to analyze P1 amplitude. The main effect is not significant for the lobe, *F*_(1,17)_ = 1.271, *p* = 0.275, η*_*p*_^2^* = 0.070, and BF_10_ = 4.296, not significant for the stimulus type, *F*_(1,17)_ = 0.292, *p* = 0.596, η*_*p*_^2^* = 0.017, and BF_10_ = 0.196, and not significant for the test number, either, *F*_(2,34)_ = 0.137, *p* = 0.872, η*_*p*_^2^* = 0.008, and BF_10_ = 0.052. Significant interaction is revealed between lobe and stimulus type, *F*_(1,17)_ = 6.883, *p* = 0.018, η*_*p*_^2^* = 0.288, and BF_10_ = 1.167. However, no significant P1 difference is suggested between regular and random patterns using the Bonferroni tests of *post hoc* in each lobe, *p*_*bonf*_ > 0.296.

No significant interaction is revealed between test ratio and stimulus type, *F*_(2,34)_ = 0.177, *p* = 0.839, η*_*p*_^2^* = 0.010, and BF_10_ = 0.111, or between test number and lobe, *F*_(2,34)_ = 2.029, *p* = 0.147, η*_*p*_^2^* = 0.107, and BF_10_ = 0.128. No significant interaction is found among the three variables, *F*_(2,34)_ = 1.161, *p* = 0.325, η*_*p*_^2^* = 0.064, and BF_10_ = 0.181.

### N1 Component

**FIGURE 7:**
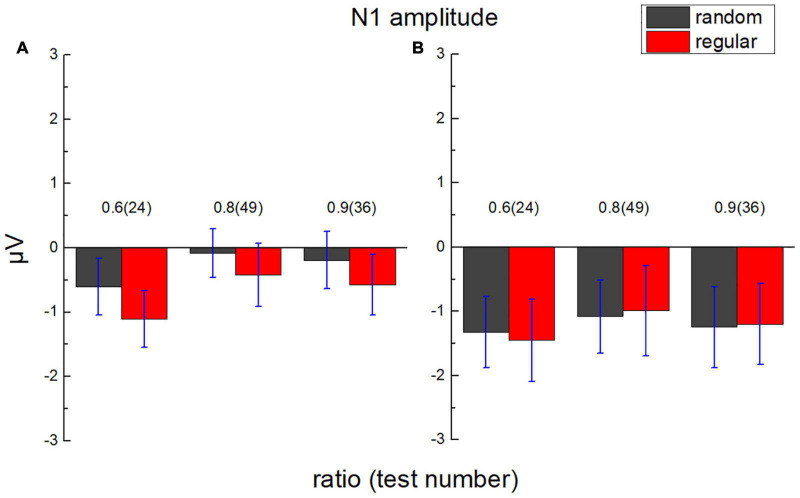
Average N1 amplitude in the parietal lobe and the occipital lobe. **(A)** The average amplitude of ERP of participants in the parietal lobe from 135 to 165 ms after test presentation (N1) is demonstrated according to different test ratios (0.6, 0.8, and 0.9). **(B)** N1 in the occipital lobe. Black bars stand for the randomly distributed patterns, and red ones stand for the regularly distributed patterns. Error bars denote 1 SE of the mean.

The average value of the ERP amplitude of participants from 135 to 165 ms after each test in the parietal and the occipital lobes was calculated, respectively, as average N1 amplitude (Figure 7). The average ERP amplitude (negative) is significantly smaller than “0,” *t*_(17)_ = 2.675, *p* = 0.008, Cohen’s *d* = 0.631, and BF_10_ = 7.107.

A 2 (lobe: parietal/occipital) × 2 (stimulus type: regular/random) × 3 (test ratio: 0.6/0.8/0.9) repeated measures ANOVA was adopted to analyze average N1 amplitude. The main effect is neither significant for the lobe, *F*_(1,17)_ = 0.837, *p* = 0.373, η*_*p*_^2^* = 0.047, and BF_10_ = 5.133, nor for the stimulus type, *F*_(1,17)_ = 1.156, *p* = 0.297, η*_*p*_^2^* = 0.064, and BF_10_ = 0.190. A significant difference is found for the test ratio, *F*_(2,34)_ = 3.834, *p* = 0.032, η*_*p*_^2^* = 0.184, and BF_10_ = 0.131. However, no significant difference is found between each two ratios using Bonferroni *Post hoc* tests, *p*_*bonf*_ > 0.074.

No significant interaction is revealed between lobe and stimulus type, *F*_(1,17)_ = 3.496, *p* = 0.079, η*_*p*_^2^* = 0.171, and BF_10_ = 0.264, between test number and lobe, *F*_(2,34)_ = 1.233, *p* = 0.304, η*_*p*_^2^* = 0.068, and BF_10_ = 0.093, or between test ratio and stimulus type, *F*_(2,34)_ = 0.182, *p* = 0.834, η*_*p*_^2^* = 0.011, and BF_10_ = 0.080. No significant interaction is found among the three variables, either, *F*_(2,34)_ = 0.018, *p* = 0.982, η*_*p*_^2^* = 0.001, and BF_10_ = 0.144.

### P2p Component

**FIGURE 8:**
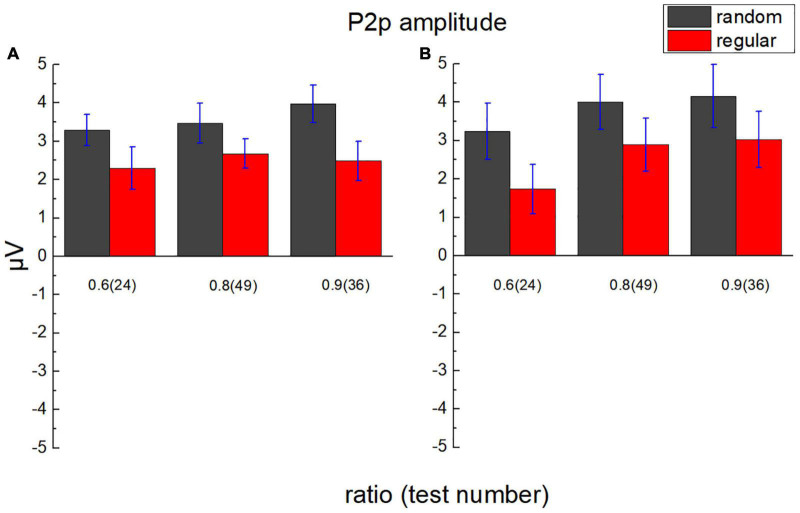
Average P2p amplitude in the parietal lobe and the occipital lobe. **(A)** The average amplitude of ERP of participants in the parietal lobe from 200 to 250 ms after test presentation (P2p) is demonstrated according to different test ratios (0.6, 0.8, and 0.9). **(B)** P2p in the occipital lobe. Black bars stand for the randomly distributed patterns, and red ones stand for the regularly distributed patterns. Error bars denote 1 SE of the mean.

The average value of the ERP amplitude of participants in the parietal lobe from 200 to 250 ms after each test (approximately 25 ms before to 25 ms after the peak of P2p) was calculated as the average P2p amplitude (Figure 8). A 2 (lobe: parietal/occipital) × 2 (stimulus type: regular/random) × 3 (test ratio: 0.6/0.8/0.9) repeated measures ANOVA was adopted to analyze P2p amplitude. The main effect is not significant for the lobe, *F*_(1,17)_ = 0.038, *p* = 0.849, η*_*p*_^2^* = 0.002, and BF_10_ = 0.002. The main effect is significant for the stimulus type, *F*_(1,17)_ = 24.417, *p* < 0.001, η*_*p*_^2^* = 0.590, and BF_10_ > 100. The average P2p amplitude activated by randomly distributed dots is significantly larger than that by regularly distributed dots, *p*_*bonf*_ < 0.001. The main effect is significant for the test number, *F*_(2,34)_ = 10.267, *p* < 0.001, η*_*p*_^2^* = 0.377, and BF_10_ = 0.780. According to Bonferroni *post hoc* tests, the P2p difference exists between ratio 0.6 and 0.8, *P*_*bonf*_ = 0.003, and it exists between ratio 0.6 and 0.9, too, *P*_*bonf*_ = 0.010. No significant difference is revealed between ratio 0.8 and 0.9, *P*_*bonf*_ = 0.990. Significant interaction is revealed between lobe and test number, *F*_(2,34)_ = 9.634, *p* < 0.001, η*_*p*_^2^* = 0.362, BF_10_ = 0.168. As is suggested by Bonferroni *post hoc* tests, in occipital area, the P2p difference is significant between ratio 0.6 and 0.8, *P*_*bonf*_ < 0.001, and it is significant between ratio 0.6 and 0.9, *P*_*bonf*_ < 0.001.

The interaction is not significant between lobe and stimulus type, *F*_(1,17)_ = 0.194, *p* = 0.665, η*_*p*_^2^* = 0.011, and BF_10_ = 0.194. The interaction is not significant between stimulus type and test ratio, either, *F*_(2,34)_ = 0.566, *p* = 0.573, η*_*p*_^2^* = 0.032, and BF_10_ = 0.010. No significant interaction is found among lobe, stimulus type, and test ratio, *F*_(2,34)_ = 3.096, *p* = 0.058, η*_*p*_^2^* = 0.154, and BF_10_ = 0.192.

No significant P2p amplitude difference is suggested between ratios 0.8 and 0.9 using Bonferroni *post hoc* tests, whereas the mean of the P2p amplitude shows a steady increase with the test ratio. To investigate whether P2p activation increases linearly with the ratio, in numerical comparison with random as well as regular patterns, linear regression was conducted with the P2p amplitude as the dependent variable and ratio as the independent variable. Two variants of models were compared, one without intercept and the other with intercept. *F*-test was performed using Origin 9 to decide which model was more reliable (Liu et al., 2020). In all conditions, the models without intercept are suggested to be better (*p* < 0.05), and the slopes are all significantly different from zero. In parietal region, for random patterns, slope = 4.58 ± 0.32 and *p* = 0.005, and for regular patterns, slope = 3.17 ± 0.29 and *p* = 0.008. In the occipital region, for random patterns, slope = 4.89 ± 0.21 and *p* = 0.002, and for regular patterns, slope = 3.35 ± 0.17 and *p* = 0.003. Noticeably, the slopes for random patterns are greater than those for regular ones, suggesting a decrease in P2p sensitivity to the number ratio for comparison in the regular group, compared with that in the random group.

**FIGURE 9:**
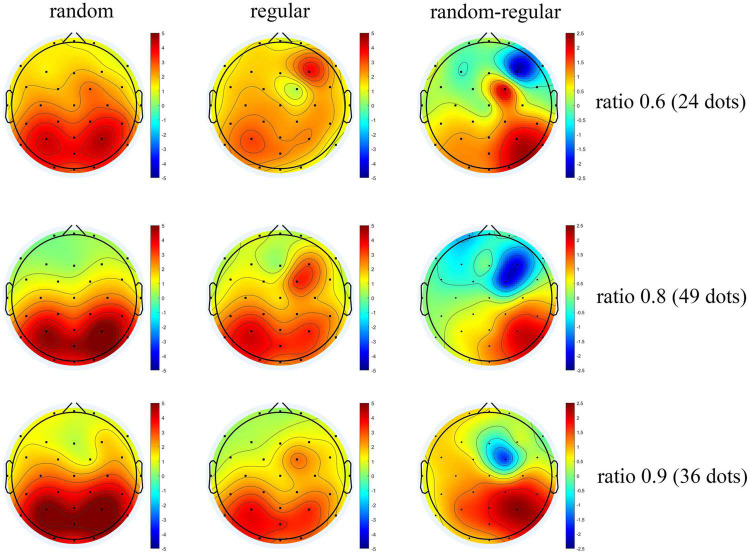
The topographic distribution of average P2p. The average amplitude of ERP of participants from 200 to 250 ms after test presentation (P2p) is shown by the scalp topography. Graphs for randomly distributed patterns are in the left column, graphs for regularly distributed patterns are in the middle column, and the subtractions of the two are shown in the right column. The graphs are arranged according to the ratios between tests and references from the upper row to the lower row.

The scalp topography is shown in Figure 9. Positive activation is revealed over the occipital-parietal area, and the activation is stronger as the ratio increases. These results are in line with previous studies (Libertus et al., 2007; Hyde and Spelke, 2009). In general, the topographic distribution of P2p in comparing regularly distributed patterns is consistent with that in comparing randomly distributed patterns. Importantly, a significant decrease is revealed over the right occipital-parietal region. According to previous studies, the non-symbolic, approximate, numerical processing is mainly carried out in the right hemisphere (Piazza et al., 2007; Holloway et al., 2010; Fornaciai and Park, 2017), suggesting that the P2p dissociation between random and regular patterns are associated with numerosity processing.

There is a noticeable activation around the cite of FC2 in the right frontal lobe. The increase might demonstrate the involvement of attention (Joseph et al., 2015). It is proposed that the density mechanism may depend on extra attention resources to analyze the local distance between adjacent dots. Attention loading could induce a higher cost for the density mechanism compared with the numerosity mechanism (Pomè et al., 2019).

## Discussion

In this study, behavioral and ERP features were analyzed for numerical comparison with random and regular patterns. Weber fraction, which estimates the precision of approximate number comparison, shows no difference between the two groups. Number distance effects on the error rate, as well as on the reaction time, are suggested in both groups. However, similar behavioral results can be mediated by distinct mechanisms. Our previous studies have demonstrated a serial of behavioral dissociation between numeral processing of random and regular patterns (Liu et al., 2017, 2018). Numerical processing with these two patterns shows distinct dependence on the distance between adjacent dots and distinct features in interocular transfer. Importantly, the connectedness effect is absent in numerical processing of regular patterns, suggesting a synchronous inhibition in the numerosity mechanism (Liu et al., 2017, 2018). In other words, the dissociation suggests a mechanism other than numerosity underlying the numeral processing with regular patterns. It is possible that the numeral comparison is achieved by comparing the distance between adjacent dots in regular patterns, and the density mechanism may supersede numerosity to compare which of the two regularly distributed dot arrays is more numerous (Liu et al., 2017, 2018). Therefore, we went further to figure out the ERP differences in numeral comparison with the regular pattern. Numeral comparison within the ANS range with randomly distributed patterns, as the task in the random group of this study, is proposed to be based on generic numerosity processing (Halberda et al., 2008; Anobile et al., 2014; Fornaciai and Park, 2017), and it is taken as a control condition.

According to the ERP analyses, early evoked components are not affected by distribution types. The P1 component is related to the early processing of object information (Luck et al., 1990). Noticeably, the random and regular patterns in the two tasks are different in some visual features, including the spatial frequency, the global shape, and the perception of a subjective configuration induced by regular distribution. Spatial frequency is proposed to be predominantly responded by the neurons in the primary visual cortex such as V1 (Issa et al., 2000). In this study, P1 is shown to be unaffected either by dot distribution pattern or by dot ratio, suggesting no difference in the early stages of processing in random and regular patterns.

N1 activation, another early evoked component, is not affected by dot distribution pattern, either. No significant N1 amplitude difference is suggested using Bonferroni *Post hoc* tests between each two ratios, in line with previous studies (Libertus et al., 2007; Hyde and Spelke, 2009, 2012). N1 is proposed to be modulated by the absolute number with small (<4) number arrays, rather than the number array beyond subitizing range (Hyde and Spelke, 2009). It is also suggested that the N1 amplitude is not modulated by the number ratio when dot arrays with a moderate number (8–30) were compared (Libertus et al., 2007).

P2p activation is modulated by the dot ratio (number distance effect), in line with previous studies (Libertus et al., 2007; Hyde and Spelke, 2009), and the distance effects are revealed both in random and regular groups. Importantly, the P2p amplitude is significantly lower for regular patterns than that for random patterns. It is controversial whether P2p is modulated by a discrete number or a continuous magnitude (Gebuis and Reynvoet, 2013; Leibovich et al., 2017). Separating numbers from all other continuous magnitudes, such as surface, density, diameter, contour length, convex hull, and so on, could be very difficult, due to the intrinsic correlations between numerosity and these magnitudes (van Rinsveld et al., 2020). The current evidence of the P2p activation difference between random and regular patterns, in which all the texture magnitudes are identical, can further demonstrate the correlation between P2p and numerosity processing from a novel perspective.

According to this study, the P2p amplitude activated by regular patterns is significantly lower than that evoked by random patterns at the same ratio and the same number, suggesting a weaker sensitivity for P2p in reflecting numerosity relationship in regular patterns. A similar decrease in sensitivity to the number ratio is also proposed by the smaller slope values of linear regression in the regular group. The decrease of sensitivity can be well explained, and it is in line with the behavioral dissociation in our previous studies (Liu et al., 2017, 2018). In those studies, regular patterns are supposed to interrupt or inhibit generic numerosity mechanism (i.e., the mechanism underlying the typical numeral comparison task, in which the dots to be compared are randomly distributed with a moderate density, Halberda et al., 2008; Anobile et al., 2014), and number comparison tasks may be facilitated by comparing the average distance of adjacent dots, rather than dot numbers, in regular patterns. Analyzing minimal distance between dots can be reliable and easy in telling which set is more numerous since this distance is identical in regular patterns with the same number of dots. Therefore, the density mechanism may take over the job to tell which of the two regular patterns contains more dots, similar to what happens in the comparison of dense patterns, in which the variation of the distance between dots decreases sharply as dots become denser, and the efficiency of perceiving mean increases (Anobile et al., 2015, 2017; Liu et al., 2017, 2018). In summary, the smaller P2p amplitude and weaker activation over the right occipital-parietal region may reflect less dependence on numerosity in the processing of regular patterns. Similar evidence suggesting that IPS activation decreases as the task puts less emphasis on quantity processing is demonstrated in a previous study (Dehaene et al., 2003).

According to a study with a passive viewing task (Fornaciai and Park, 2017), weaker P2p sensitivity to numerosity is revealed within a very large number range, compared with that within moderate or ANS range, over right occipito-parietal cites. The current results, which demonstrate that P2p for the processing of regular patterns is less activated and is less sensitive to the number ratio, is consistent with this study. In this study, significant positive activation is shown in the occipital and parietal lobes for both groups. The difference between random and regular conditions is revealed mainly in the same regions, especially in the right occipito-parietal region. This region is associated with non-symbolic approximate numeral processing (Fornaciai and Park, 2017); therefore, it is proposed that the dissociation in processing with random and regular patterns is highly correlated with numerosity.

The related lobes of numerosity processing have been investigated since the 1980s. Functional magnetic resonance imaging (fMRI) study found that the bilateral occipital lobe and prefrontal cortex (PFC) are activated during numeral calculating procedure (Roland and Friberg, 1985; Dehaene, 1996). The IPS is related to numeral representation, and the activation in this area is independent of the format (symbolic or non-symbolic) of numeral information (Piazza et al., 2007). IPS is also revealed to be activated in passive tasks, in which participants watched the numeral stimuli without being asked for any response (Ansari et al., 2006). In this study, the topographic distribution and the latency of P2p in comparing random and regular patterns are similar, and a significant decrease is mainly revealed in the right occipital-parietal lobes in the regular group. It is worth noting that IPS is proposed to be specific to a number change, rather than a shape or symbol change of stimuli (Piazza et al., 2004; Harvey et al., 2013). It is unlikely that the decrease in these regions can be attributed to shape differences between random and regular patterns.

Nevertheless, we still need to rule out the possibility that P2p amplitude differences found in this study are due to the difference in stimuli configuration, rather than the distinct numeral mechanisms induced by different dot distributions. An important aspect of shape processing is the sensitivity to “good continuation” induced by the collinearity of local elements (Kourtzi et al., 2003). In this study, a better-defined configuration (i.e., a matrix) can automatically emerge from regularly distributed dot arrays, and therefore, the perception of the subjective contour may contribute to the P2p difference between random and regular groups. This potential explanation is challenged from some perspectives. A previous study, which aimed at comparing the fMRI activation in perceiving subjective configuration with that in perceiving random patterns, proposed that the detection of collinear contour induces a stronger activity of neurons not only in higher visual areas but also in early areas such as V1. The selectivity to collinear contours, compared with random patterns, was observed in the bilateral V1–V4, the lateral occipital complex (LOC), and the temporal lobe (Kourtzi et al., 2003). In this study, similar dot patterns were adopted with a subjective contour emerging from regular distribution. According to the configuration explanation, one would have expected ERP differences between patterns in early components such as P1 and/or N1, differences between patterns mainly in the occipital region, and a greater rather than smaller amplitude of P2p in the regular group compared with that in the random group because the pattern with subjective contour can induce stronger neuron activity, compared with random patterns (Kourtzi et al., 2003). However, the current results are in contrast with these hypotheses. Noticeably, no difference is found between patterns in early evoked P1 and N1 components. Differences in P2p are revealed in both occipital and parietal regions, especially over the right occipital-parietal region, which is associated with non-symbolic approximate numeral processing (Piazza et al., 2007; Holloway et al., 2010; Fornaciai and Park, 2017). Moreover, the P2p amplitudes are significantly smaller, rather than greater, for regular patterns, than those for random patterns.

In summary, the current results cannot be attributed to configuration differences. In contrast, the greater P2p amplitude, the higher sensitivity of P2p response to the number ratio, and the involvement of the right occipital-parietal region in revealing P2p difference between the two groups can be well explained by the hypothesis that compared with processing with regular patterns, numerical processing with random patterns triggers the mechanism of numerosity. Therefore, the increase in both P2p-amplitude and P2p-sensitivity to the ratio is observed, and the identical region that underlined the processing difference between numerosity and density mechanisms (Fornaciai and Park, 2017) is revealed repeatedly in this study. In addition, the correlation between P2p and numerosity processing is emphasized in this study. Most continuous magnitudes, such as surface, density, diameter, and convex hall, are identical in random and regular patterns. Still, differences in P2p amplitudes are revealed when a mechanism other than numerosity is adopted in numeral tasks with regular patterns. More studies are provided, supporting the view that P2p activation is mediated by numerosity processing, rather than merely induced by continuous magnitude processing.

## Data Availability Statement

The original contributions presented in the study are included in the article/supplementary material, further inquiries can be directed to the corresponding author.

## Ethics Statement

The studies involving human participants were reviewed and approved by the Ethics Committee of the Zhejiang University. The patients/participants provided their written informed consent to participate in this study.

## Author Contributions

ZZ and WL developed the study concept and contributed to the study design. YF performed the testing and data collection and provided the critical revisions. YF and LW performed the data analysis and interpretation under the supervision of ZZ. WL drafted the manuscript. CW drew the figures. All authors contributed to manuscript revision, read, and approved the submitted version.

## Conflict of Interest

The authors declare that the research was conducted in the absence of any commercial or financial relationships that could be construed as a potential conflict of interest.

## Publisher’s Note

All claims expressed in this article are solely those of the authors and do not necessarily represent those of their affiliated organizations, or those of the publisher, the editors and the reviewers. Any product that may be evaluated in this article, or claim that may be made by its manufacturer, is not guaranteed or endorsed by the publisher.

## Footnotes

1
http://www.bootstrap-software.com/psignifit/
